# Automatic Segmentation of Periapical Radiograph Using Color Histogram and Machine Learning for Osteoporosis Detection

**DOI:** 10.1155/2023/6662911

**Published:** 2023-02-28

**Authors:** Rini Widyaningrum, Enny Itje Sela, Reza Pulungan, Anindita Septiarini

**Affiliations:** ^1^Department of Dentomaxillofacial Radiology, Faculty of Dentistry, Universitas Gadjah Mada, Yogyakarta 55281, Indonesia; ^2^Department of Informatics, University of Technology of Yogyakarta, Yogyakarta 55285, Indonesia; ^3^Department of Computer Science and Electronics, Universitas Gadjah Mada, Yogyakarta 55281, Indonesia; ^4^Department of Informatics, Mulawarman University, Samarinda 75119, Indonesia

## Abstract

Osteoporosis leads to the loss of cortical thickness, a decrease in bone mineral density (BMD), deterioration in the size of trabeculae, and an increased risk of fractures. Changes in trabecular bone due to osteoporosis can be observed on periapical radiographs, which are widely used in dental practice. This study proposes an automatic trabecular bone segmentation method for detecting osteoporosis using a color histogram and machine learning (ML), based on 120 regions of interest (ROI) on periapical radiographs, and divided into 60 training and 42 testing datasets. The diagnosis of osteoporosis is based on BMD as evaluated by dual X-ray absorptiometry. The proposed method comprises five stages: the obtaining of ROI images, conversion to grayscale, color histogram segmentation, extraction of pixel distribution, and performance evaluation of the ML classifier. For trabecular bone segmentation, we compare K-means and Fuzzy C-means. The distribution of pixels obtained from the K-means and Fuzzy C-means segmentation was used to detect osteoporosis using three ML methods: decision tree, naive Bayes, and multilayer perceptron. The testing dataset was used to obtain the results in this study. Based on the performance evaluation of the K-means and Fuzzy C-means segmentation methods combined with 3 ML, the osteoporosis detection method with the best diagnostic performance was K-means segmentation combined with a multilayer perceptron classifier, with accuracy, specificity, and sensitivity of 90.48%, 90.90%, and 90.00%, respectively. The high accuracy of this study indicates that the proposed method provides a significant contribution to the detection of osteoporosis in the field of medical and dental image analysis.

## 1. Introduction

Nowadays, the use of dental radiographs is no longer limited to assessing dental problems. Another thriving application that utilizes dental radiographic images is osteoporosis detection [1, 2]. Osteoporosis is a metabolic bone disorder characterized by the loss of cortical thickness and the reduced number and size of trabeculae [3, 4]. Risk factors that can cause osteoporosis include age, gender, and life activities. Osteoporotic fractures have been the most significant complication of osteoporosis [5] that subsequently increases medical costs, mortality, and morbidity, especially among the elderly population. It is also known that osteoporosis has a direct effect on the progression of periodontal tissue destruction [6], tooth loss, and erosion of the jaw bones [3]. Therefore, dental radiography can be recommended as a screening tool for osteoporosis [1, 3, 6].

The role of the dentist is pivotal in the early detection of osteoporosis on dental radiographs. However, manual detection of osteoporosis on dental radiographs depends on the experience of the dentist and oral radiologist. Conducting training for using dental radiographs for early detection of osteoporosis will take a lot of time. The automation of radiographic interpretation procedures could assist dentists' decision-making while also saving time and effort [7]. For these reasons, automatic detection of osteoporosis on a periapical radiograph becomes important to assist dentists, particularly in overcoming misinterpretation due to fatigue or lack of experience, as well as in conducting the detection in less time.

With the advancement of machine learning (ML) and pattern recognition methods, dental radiographs are increasingly used for osteoporosis detection. Recent methods have overcome the lack of quality of radiographic images, including low contrast, significant noise, and color homogeneity in the regions of interest (ROI). Several image processing methods have been investigated to detect osteoporosis using dental radiographs, including periapical [8, 9] and panoramic [2] radiographs, as well as CBCT [10]. Another method, finite element analysis combined with ML, is used to predict hip fracture in DEXA images [11].

Image segmentation is used to divide digital images into multiple sets of pixels or objects so that image representation becomes more straightforward and easier to analyze. However, in the absence of a conventional solution to image segmentation, researchers have proposed general-purpose techniques and algorithms to implement image segmentation. These techniques and algorithms often need to be combined with domain knowledge to effectively solve the image segmentation problem in a specific domain [12]. Segmentation is a crucial process for medical image analysis, radiological assessment, classification, and computer-aided diagnostic systems [13].

Image segmentation methods can be classified into three categories: edge-based, region-based, and pixel-based methods [14]. Clustering is a pixel-based segmentation method and is usually used for large-sized images [15]. Since it is pixel-based, clustering involves relatively simple algorithms, and its complexity is generally lower than that of region- or edge-based segmentation methods. The performance of clustering algorithms for image segmentation is susceptible to the features of objects in the image [16]. Furthermore, clustering is suitable for biomedical image segmentation.

Several basic clustering algorithms have been used for medical image segmentation, including Fuzzy clustering for dental radiographs [17] and K*-*means clustering to detect breast cancer on digital mammograms [18]. Clustering is a method of unsupervised learning used to find structures in datasets that are not labeled. A cluster is a collection of similar objects that are different from objects belonging to other clusters [19]. K*-*means is a simple technique for data clustering. In simple terms, K*-*means is an algorithm for grouping objects based on specific features into K groups, where K is a positive integer. Grouping analysis aims to group objects so that similar objects reside in the same cluster. Each cluster is characterized by its center point, e.g., its centroid. Grouping is carried out by minimizing the distance between each object and its corresponding centroid. Fuzzy C-means, on the other hand, is a soft clustering technique in which each pixel can belong to two or more clusters with varying degrees of membership. The closer the data is to the cluster center, the greater its membership in that cluster center. Despite the fact that it only considers image intensity values and no filtering, it is highly resistant to noise and offers better segmentation quality [20].

This study proposed a pixel-based clustering segmentation method on periapical radiographs using K*-*means and Fuzzy C-means. Color images are generated by the segmentation models and the separation of trabecular bone and porous was performed using K*-*means and Fuzzy C-means for grouping objects of the same color. To detect osteoporosis, three supervised ML classifiers were used: decision tree, naive Bayes, and multilayer perceptron.

The decision tree and naive Bayes have been evaluated for automated detection of carious lesions in photographic color tooth images [21]. As an osteoporosis detection model, the performance of the decision tree on panoramic radiographs is excellent [22]. In previous research, naive Bayes was investigated as a ML algorithm for evaluating canine impaction on radiographic images [23]. Naive Bayes is selected because it performs optimally on simple and small training datasets, and because our features are independent of one another. Furthermore, multilayer perceptron is a type of feedforward artificial neural network with full connectivity that generates a set of outputs from a set of inputs. This ML algorithm is characterized by multiple layers of input nodes connected as a directed graph between the input and output layers [24]. The multilayer perceptron with backpropagation has been used in a wide range of applications, including optical character recognition and medical image analysis. Since periapical radiographs are a useful tool for predicting osteoporosis [9], we are interested in exploring these ML algorithms (decision tree, naive Bayes, and multilayer perceptron) as a classifier in this study. We hypothesize that the proposed method of color histogram of pixel-based clustering segmentation combined with an ML classifier can improve the diagnostic performance of osteoporosis on digital periapical radiographs.

## 2. Materials and Methods

### 2.1. Materials

The study used 102 digital periapical radiographs from the anterior and posterior regions of the mandible, collected from postmenopausal Javanese women (aged 58–81 years). The samples were obtained retrospectively from the dental hospital at Universitas Gadjah Mada. Ethical approval was achieved from the local ethics committee (Ref. No. 00681/KKEP/FKG-UGM/EC/2016). Data of patients were kept anonymous.

The periapical radiographs were taken using an X-ray dental machine Villa System Medical Endos ACP CEI, with an exposure specification of 70 kVp, 8 mA, and the exposure time was varying from 0.02 seconds to a maximum of 3.20 seconds. Vista Scan photostimulable phosphor (PSP) plates were used as image receptors. The periapical images were processed using indirect digital radiography (DBSWin 4.5). All periapical radiographs involved in this study have passed the radiographic quality assurance standards assessed by a dentist based on the quality rating criteria for digital images [25]. In this study, only radiographs that meet the minimum diagnostically acceptable criteria have been taken as samples. Initial images obtained from the digital radiographic system were 1645 pixels in height and 1252 pixels in width with indexed color type and saved as BMP format.

The diagnosis of osteoporosis was obtained from the medical record. The osteoporosis diagnosis was assessed using the T-score value obtained from the DEXA scan using the Lunar Prodigy Primo DEXA densitometer, specification 76 kV, 1.5 mA, with exposure 14 seconds (femoral) and 1 minute 27 seconds (spine). The results of the DEXA scan were interpreted by a radiologist based on the definition of osteoporosis by the World Health Organization *T* score, which divides the status into three categories, i.e., normal (*T* ≥ −1.0), osteopenia (−2.5 < *T* < −1.0) and osteoporosis (*T* ≤ −2.5). According to prior studies [22], the normal and osteopenia were categorized in nonosteoporosis data in this study. The data used in this study consist of 52 nonosteoporosis (30 images for training and 22 images for testing) and 50 osteoporosis subjects (30 images for training and 20 images for testing).

### 2.2. Methods

**Figure 1:**
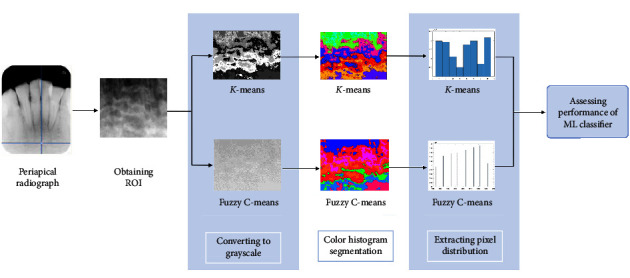
The proposed method for automatic segmentation to detect osteoporosis on periapical radiographs.

This work proposed a method for automatic segmentation of the trabecular bone area in periapical radiographs for osteoporosis detection. The proposed method consists of five main stages, i.e., obtaining ROI images, conversion to grayscale, color histogram segmentation, extraction of pixel distribution, and evaluation of the ML classifier's performance. These steps are shown in Figure 1, and the details of each stage are explained in the following.

#### 2.2.1. Obtaining ROI Images

**Figure 2:**
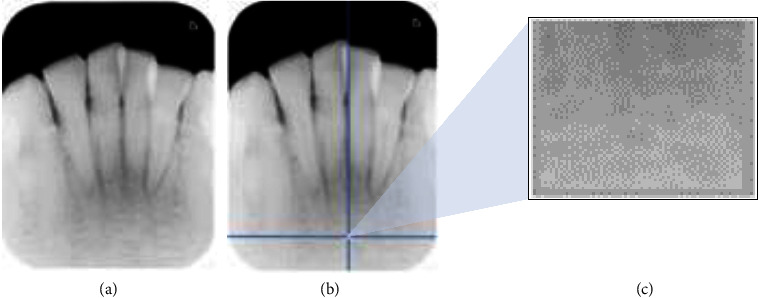
ROI selection: (a) original image, (b) starting point for ROI, and (c) ROI.

The initial stage of the study involves the acquisition of ROI images from periapical radiographs. In this work, ROI is obtained by our software that has been developed using MATLAB, as described in a prior study [26]. The process of determining ROI begins with selecting a starting point in the trabecular area visible on periapical radiographs, which is taken a minimum 2 mm from the apical of the teeth and determined by avoiding tooth roots, periapical or intraosseous lesions, or variations that affect normal trabecular conditions. A square ROI can be formed from this point, measuring 300 pixels in height and 400 pixels in width (Figure 2). The ROI selection was made under the supervision of a dentist.

#### 2.2.2. Converting to Grayscale

In medical image analysis, it is common to convert the images to grayscale. ROI images were then transformed into grayscale so that each pixel in the image has a value in the range of 0– 255. Grayscale images are then used as inputs for segmentation analysis.

#### 2.2.3. Segmentation

ROI consists of trabecular (cancellous) bone and pores. The cancellous bone consists of a structural mesh of trabeculae form. Porous is the presence of small holes in the trabecular bone. By combining the color histogram and K*-*means algorithm for segmentation, the same color is assigned to pixels with the same centroid to obtain segmented images at the end of the segmentation stage [20].

We also compared the results of K*-*means with those using the Fuzzy C-means segmentation method. Fuzzy C-means is an unsupervised extension of K*-*means, applied to various problems related to the analysis of features and the design of the classifier. In this study, the Fuzzy C-means segmentation method refers to the modified Fuzzy C-means method developed in the previous study [27].

#### 2.2.4. Extracting Pixel Distribution

At this stage, the features are derived from the segmented images by calculating the distribution of the same-colored pixels. For each color, the number of pixels is counted and represented in a histogram. The histogram is a summary bar graph showing the frequency of data points falling in various ranges. All features are stored in a DAT format file.

#### 2.2.5. Assessing Classifiers' Performance

The final stage of osteoporosis detection is evaluating the performance of the classifier. There are two processes at this stage, i.e., training and testing. Both training and testing use the features constructed in the previous stage (the number of pixels in each group) as inputs to the osteoporosis classification process. The training process was used to determine the best classifier model for the testing process. On the other hand, the testing process is used to decide on each test dataset, whether it belongs to the nonosteoporosis or osteoporosis classes. The number of ROI images used in training was 60 images (30 images of nonosteoporosis class and 30 images of osteoporosis class) and the rest of the image datasets were used for testing (42 images: 22 images of nonosteoporosis class and 20 images of osteoporosis class).

To obtain the optimal results, we conducted three ML methods: decision tree (J48 algorithm), naive Bayes, and multilayer perceptron. The parameters of the multilayer perceptron applied in this study were the number of hidden layers is 2, the activation function is sigmoid, the learning rate is 0.3, and the momentum is 0.2.

The performance of the proposed method in this study was assessed based on accuracy, specificity, and sensitivity. Evaluation of the classifier's performance was conducted in two stages, on training and testing datasets, to determine the most appropriate algorithm for predicting osteoporosis using periapical radiograph features in this study. Due to the fact that each of the two segmentations (K-means and Fuzzy C-means) is evaluated using three ML classifiers, a total of six algorithms are evaluated using the training dataset. The algorithm with the highest performance evaluation on the training dataset was then reevaluated using the testing dataset. The final result of this study was determined by the performance evaluation on the testing dataset.

## 3. Results

By applying the K*-*means method to ROI images, this study has produced segmentation images in grayscale and color formats as well as their histograms. The method's performance has been validated for accuracy, specificity, and sensitivity based on the features of the number of clustering pixels in the histogram.  Table 1 shows the examples of grayscale and color segmentation results using the K*-*means method applied to a similar ROI image. Meanwhile, Table 2 displays the results of image segmentation using Fuzzy C-means.

Since the grayscale segmentation images had almost similar colors, the cluster areas were still difficult to recognize. Therefore, they are converted into color segmentation images. A grayscale segmentation image corresponds precisely to a specific color in the corresponding color segmentation image.

Tables 1 and 2 also show the histograms of the segmentation images. The histogram's *x*-axis represents the number of cluster sequences formed in the segmentation with *k* = 8, 10, 12, and 15, while the *y*-axis represents the number of pixels in each cluster. We compare the results of the K*-*means segmentation algorithm (Table 1) with Fuzzy C*-*means segmentation (Table 2).

The grayscale images segmented by Fuzzy C-means in Table 2 have less contrast than the K-means segmented images in Table 1. Therefore, the color segmentation images produced by K-means (Table 1) also differ from those produced by Fuzzy C-means (Table 2). To determine whether the differences in K-means segmentation (Table 1) and Fuzzy C-means segmentation (Table 2) influence osteoporosis detection, the performance of the training using the two segmentation methods is then evaluated, with a summary of testing results presented in Table 3.

The performance of the training was evaluated by comparing the actual osteoporosis diagnosis results from the DEXA examination and the predicted results using the K*-*means and Fuzzy C-means segmentation methods, with the pixel distribution in each cluster serving as the feature. For *K* = 8, 10, 12, and 15, the sizes of the features produced were 102 × 8, 102 × 10, 102 × 12, and 102 × 15, respectively. These features are then tested using three classifiers: decision tree, naive Bayes, and multilayer perceptron.

Using a confusion matrix, the performance of classifiers in the training process is assessed based on three characteristics: accuracy, specificity, and sensitivity. Accuracy, specificity, and sensitivity are presented in percentages. Therefore, if the segmentation results of the proposed method is perfect; its accuracy must be close to 100%. The performance of training using the K*-*means classification in comparison to Fuzzy C-means classification is presented in Table 3.

According to Table 3, the global performance of K-means segmentation (81.67%) is higher compared to that of Fuzzy C-means segmentation (75.28%). Among the three ML classifier methods used to evaluate K*-*means and Fuzzy C-means segmentation, the multilayer perceptron produces the highest average performance, followed by the decision tree for K*-*means segmentation and naive Bayes for Fuzzy C-means segmentation. For K-means segmentation, the diagnostic performance of naive Bayes for osteoporosis was the lowest. Meanwhile, decision tree provides the lowest performance for osteoporosis detection using the Fuzzy C-means segmentation (Table 3).

Based on the training performance in Table 3, The K*-*means combined with the multilayer perceptron model successfully detects osteoporosis and nonosteoporosis conditions with the highest accuracy, specificity, and sensitivity of 91.67%, 90.00%, and 93.33%, respectively. It was obtained from the K*-*means segmentation with *K* = 10 combined with a multilayer perceptron classifier, using only a feature in the form of the number of constituent pixels of each cluster. In addition, the algorithm proposed in this study can rapidly detect osteoporosis. It takes less than a minute to obtain the detection result, counting from taking the ROI in the periapical radiograph.

Since this study aims to propose an automatic detection of osteoporosis using periapical radiographic images, the model with the best training performance was then being tested using 42 ROI images. The performance of testing is presented in Table 4 as a confusion matrix. The testing results shown in Table 4 indicates that the segmentation method using the K*-*means (*K* = 10) combined with multilayer perceptron classifier is the most appropriate method for the proposed method, which achieves accuracy, specificity, and sensitivity of 90.48%, 90.90%, and 90.00%, respectively.

## 4. Discussion

Periapical and other dental radiograph images can be noisy, blurred, and dark. Due to these issues, conventional segmentation methods for osteoporosis detection are ineffective. Several studies on osteoporosis detection using image segmentation reported higher accuracy [10, 28] than studies without segmentation [4, 5].

Several studies have been conducted to obtain important features for detecting osteoporosis using image segmentation on dental radiographs combined with ML. Using support vector machine (SVM) multiclass, a previous study [4] classified data into normal bone, osteopenia, and osteoporosis. Categorization was established through an examination of alterations in the trabecular pattern of the mandible bone. The image was enhanced using adaptive histogram equalization and the gray level co-occurrence matrix (GLCM) was used to extract features. Texture analysis was also used to detect osteoporosis on panoramic radiographs, followed by logistic regression, which served as a classifier and was quantified using the receiver operating characteristic of the receiver [5].

A previous study also used a combination of morphology analysis, fractal dimensions, GLCM, and SVM to detect osteoporosis using periapical radiographs. On 454 panoramic radiographs, the fractal dimension was segmented using Gaussian blur, density correction, binarization, and skeletonization [22]. Otsu segmentation and canny edge detection are also known for extracting seven features: trabecular termini, trabecular separation, trabecular spacing, trabecular number, and trabecular thickness. The classifier was implemented using the backpropagation of an artificial neural network [10]. In several studies [10, 22, 29], segmentation was also performed using morphological analysis. However, objects' shapes may change, rendering the extracted features ineffective.

In this work, an osteoporosis classification method for dental periapical radiographic images has been developed using a pixel distribution based on a segmentation approach using the K*-*means followed by a decision tree, naive Bayes, and multilayer perceptron classifier. The result was obtained after we compared the K*-*means segmentation method's performance on periapical radiographs with other existing segmentation methods, e.g., Fuzzy C-means. Fuzzy C-means have been widely used in medical diagnosis, image analysis, shape analysis, and target recognition [27]. Our experimental results using the K*-*means revealed that the method is more accurate than Fuzzy C-means (Table 3) for osteoporosis detection on periapical radiograph.

Image segmentation is the process of breaking down or partitioning an image into separate homogeneous regions in order to make the image easier to analyze and identify objects. During this activity, the object and the background are separated [12]. In addition to edge-based and region-based segmentation, the results of this study indicate that pixel-based segmentation in the form of K*-*means and Fuzzy C-means is more suitable for the analysis of osteoporosis-related trabecular changes on periapical radiographs. Considering that image segmentation is one of the most fundamental steps in image processing, selecting the appropriate segmentation method will undoubtedly impact the diagnostic performance of the detection model.

Naive Bayes is known as the osteoporosis classifier for textural features extracted from panoramic radiographs utilizing fractal dimension (FD) and GLCM [30]. In addition to naive Bayes, decision trees, and SVM have been found to be ML classifiers with superior performance when applied to detect osteoporosis using strut analysis, FD, and GLCM extracted from mandibular cortical bone on panoramic radiographs [22]. For the detection of osteoporosis using periapical radiographs, neither the naive Bayes nor decision tree methods have been extensively studied.

Despite naive Bayes' optimal performance for a simple and small training datasets, its performance in this study is inferior to that of prior studies [30]. Additionally, the diagnostic performance of the decision tree for osteoporosis detection using periapical radiographs in the study does not outperform earlier research using panoramic radiographs [22]. The segmentation methods used in this study differ from those used in previous studies [22, 30], in addition to using distinct ROI obtained from different radiograph types. On panoramic radiographs, osteoporosis is generally detected by observing the thickness of the mandibular cortex, whereas osteoporosis is detected on periapical radiographs by observing the changes of bone trabeculation in the mandible and maxilla.

In general, the segmentation technique is followed by classification, in which the separated objects are assigned to specific classes, and this process is regarded as the fundamental part of computer vision [12]. At the end of the segmentation in this study, a histogram of the clustering was produced. The histogram represents the feature, which is the number of pixels in a cluster. For validation, the study evaluated the segmentation performance of K*-*means based on three measurements: accuracy, specificity, and sensitivity. We observed various *k* values (*k* = 1 up to 15) and reported only the accuracy for each classifier method resulting more than 60% (Table 3).

To further evaluate the proposed method's performance, we also compared the proposed method's results with other methods based on previous studies. In this study, histogram extraction was used to produce relatively simpler features than in other studies. Besides, the number of classes used in this research study is also more than in other studies. In addition, the method we proposed reached a testing accuracy of 90.48% for osteoporosis and nonosteoporosis classes. This performance is better than the previous studies [4, 5, 29, 31] but slightly worse than previous works [10, 22].

Based on the results of the study (Tables 3 and 4), the multilayer perceptron provides the best performance as a classifier for image features generated by the color histogram of pixel-based clustering segmentation. This ML classifier performs best when combined with K*-*means segmentation for predicting osteoporosis on periapical radiographs. These findings support our previous studies [31] while also demonstrating that multilayer perceptron classification of osteoporosis on periapical radiographs performs better when combined with K*-*means segmentation than segmentation using a texture feature with a statistical approach.

We presume that factors related to the number of samples and the method of feature extraction may affect the accuracy of the results. Despite the fact that this method has never been applied to periapical radiographs to detect osteoporosis, the results of this study indicate that it has a high diagnostic performance.

## 5. Conclusion

K*-*means outperforms Fuzzy C-means for segmenting trabecular bone observed on periapical radiographs, especially when combined with multilayer perceptron as a ML classifier for osteoporosis detection. However, this study has drawbacks due to the limited number of samples. It is challenging to obtain periapical radiographs from patients who have undergone a DEXA examination. Future work with larger samples can be conducted so that the findings can be generalized to large populations. The result of this study indicates that the proposed method promotes a valuable contribution to the medical image analysis for osteoporosis detection that can be further developed for dental practice application.

## Figures and Tables

**Table 1:** The results of segmentation using K*-*means.

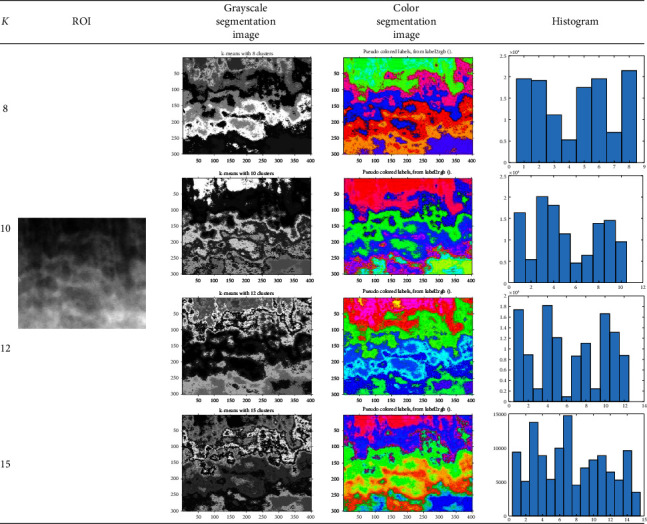

**Table 2:** The results of segmentation using Fuzzy C-means.

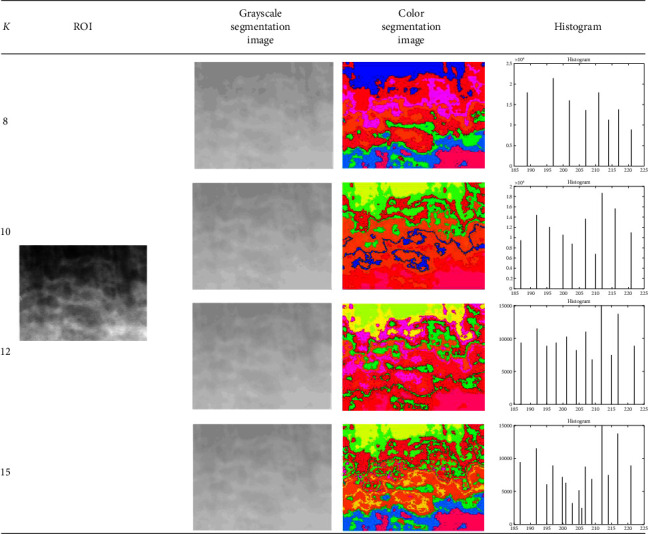

**Table 3:** Training performance of K*-*means and Fuzzy C-means classification.

Segmentation	ML classifier method	Performance	*K*				Average performance^*∗*^	Global performance^*∗∗*^
			8	10	12	15		
K*-*means	Decision tree	Accuracy	86.67	88.33	80	75	82.50	81.67
		Specificity	83.33	90	76.67	73.33		
		Sensitivity	90	86.67	83.33	76.67		
	Naive Bayes	Accuracy	75	83.33	76.67	73.33	77.08	
		Specificity	73.33	80	80	66.67		
		Sensitivity	76.67	86.67	73.33	80		
	Multilayer perceptron	Accuracy	88.33	91.67	80	81.67	85.42	
		Specificity	86.67	90	76.67	83.33		
		Sensitivity	90	93.33	83.33	80		

Fuzzy C-means	Decision tree	Accuracy	78.33	75	63.33	71.67	72.08	75.28
		Specificity	83.33	70	70	76.67		
		Sensitivity	73.33	80	56.67	66.67		
	Naive Bayes	Accuracy	80	70	73.33	73.33	74.17	
		Specificity	80	73.33	63.33	70		
		Sensitivity	80	66.67	83.33	76.67		
	Multilayer perceptron	Accuracy	81.67	68.33	83.33	85	79.58	
		Specificity	83.33	70	80	83.33		
		Sensitivity	80	66.67	86.67	86.67		

^*∗*^The average performance describes the average of all performance characteristics (accuracy, specificity, or sensitivity) of an ML classifier. ^*∗∗*^The global performance is derived from the average of all parameters (accuracy, specificity, and sensitivity) of segmentation methods utilizing all ML classifiers.

**Table 4:** Testing's performance.

Prediction	Actual		
	Osteoporosis	Nonosteoporosis	Total
Osteoporosis	18	2	20
Nonosteoporosis	2	20	22
Total	20	22	42

## Data Availability

The data supporting the findings of the study are included in the manuscript.
